# Supplementary material for: NDP52 mediates an antiviral response to hepatitis B virus infection through Rab9-dependent lysosomal degradation pathway
Source: Nat Commun. 2023 Dec 19;14:8440. doi: 10.1038/s41467-023-44201-2 (PMC10730550; doi:10.1038/s41467-023-44201-2)

SUPPLEMENTARY INFORMATION

for

NDP52 mediates an antiviral response to hepatitis B virus infection through Rab9-dependent lysosomal degradation pathway

Shuzhi Cui[1], Tian Xia[1,2], Jianjin Zhao[1], Xiaoyu Ren[1], Tingtao Wu[1], Mireille Kameni[1], Xiaoju Guo[1], Li He[1], Jingao Guo[1], Aléria Duperray-Susini[2], Florence Levillayer[2], Jean-Marc Collard[1,2], Jin Zhong[1], Lifeng Pan[3], Frédéric Tangy[2], Pierre-Olivier Vidalain[2,4], Dongming Zhou[5], Yaming Jiu[1], Mathias Faure[4] and Yu Wei[1,2*]

[1] University of Chinese Academy of Sciences, Chinese Academy of Sciences, 320 Yueyang Road, 200031 Shanghai, China
[2] Institut Pasteur, Université Paris Cité, 28 rue du Dr. Roux, 75015 Paris, France
[3] Shanghai Institute of Organic Chemistry, Chinese Academy of Sciences, 345 Lingling Road, 200032 Shanghai, China
[4] CIRI, Centre International de Recherche en Infectiologie, Univ Lyon, INSERM U1111, CNRS UMR5308, Université Claude Bernard Lyon 1, Ecole Normale Supérieure de Lyon, 69007 Lyon, France
[5] Department of Pathogen Biology, School of Basic Medical Sciences, Tianjin Medical University, 300070 Tianjin, China
*Corresponding author : Yu Wei, Institut Pasteur, 28 rue du Dr. Roux, 75015 Paris, France. Phone : (33)145688862, email : yu.wei@pasteur.fr

a

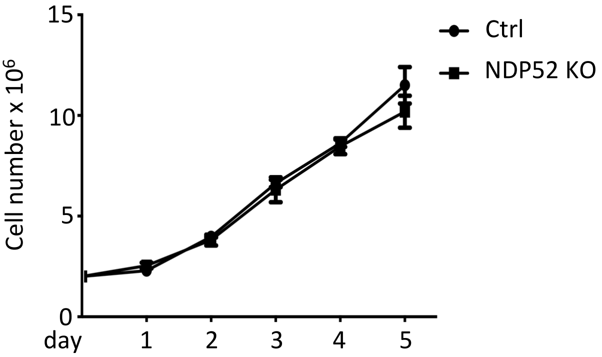

b

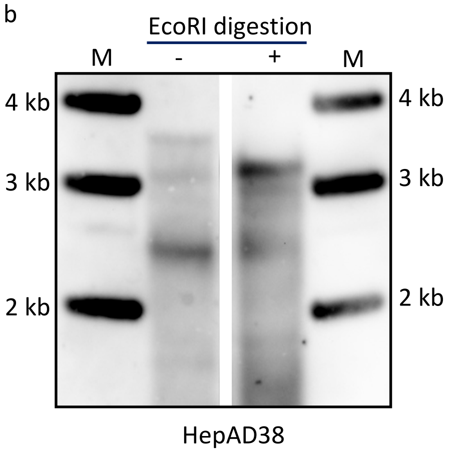

Supplementary Fig. 1. Knockout of NDP52 does not have effect on cell growth. a, Equal numbers of CRISPR control NDP52$^{HepG2\text{-}NTCPWT}$ (Ctrl) and NDP52$^{HepG2\text{-}NTCPKO}$ (NDP52 KO) cells were plated in triplicate. Cell numbers were determined during 5 days (n = 2 biological replicates). The data presented are the mean value $\pm$ S.D. obtained from three independent experiments. **b,** Example of HBV DNA replicative forms detected by Southern blot analysis. HepAD38 cells were collected seven days after doxycycline removal. Cytoplasmic core DNA was purified and was subjected to EcoRI digestion, which linearizes viral relaxed circular genome. Viral species were detected by Southern blot analysis using an HBV DNA probe. M: DNA marker. Source data for a are provided as Source Data file. Source data for b are provided as supplementary information.

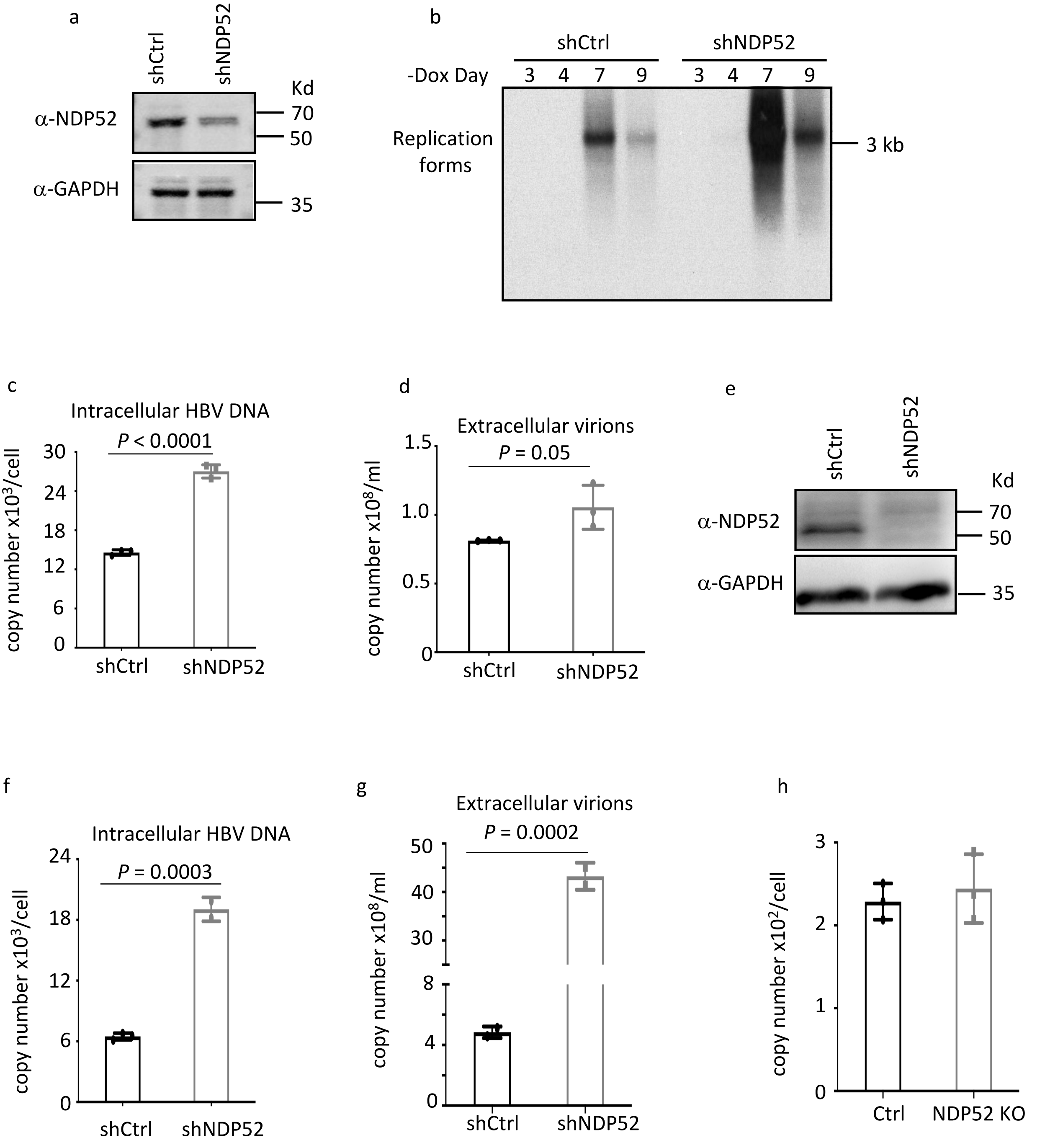

Supplementary Fig. 2. Knockdown of NDP52 in HepAD38 and Huh7 cells increases HBV replication. a, Immunoblot analysis of indicated proteins from cellular extracts of *NDP52*-knockdown HepAD38 (shNDP52) and control cells (shCtrl). **b**, HepAD38 cells were collected at indicated time after doxycycline removal. Cytoplasmic core DNA was purified and detected by Southern blot analysis using an HBV DNA probe. **c**, **d**, Doxycycline was removed from shCtrl or shNDP52 HepAD38 cells. Seven days later, HBV DNA from cells (c) or extracellular virions (d) were analyzed by qPCR (n = 3 biological replicates). **e**, Immunoblot analysis of indicated proteins from cellular extract of *NDP52*-knockdown Huh7 cells (shNDP52) and control cell line (shCtrl). **f**, **g**, shCtrl or shNDP52 Huh7 cells were transfected with an overlength HBV genomic construct competent to initiate viral replication cycle. Four days later, HBV DNA from cells (f) or extracellular virions (g) were analyzed by qPCR (n = 2 biological replicates). **h**, Attachment assay in $NDP52^{HepG2\text{-}NTCPWT}$ (Ctrl) and $NDP52^{HepG2\text{-}NTCPKO}$ (KO) cells. HBV particles were incubated with cells for 2 h. Cells were lysed to extract DNA. Viral DNA was analyzed by qPCR (n = 3 biological replicates). Data are means ± SD. Statistical significance in (c, d, f, g) is determined by a two-sided unpaired t-test. Source data for c,d,f,g,h are provided as Source Data file. Source data for a,b,e are provided as supplementary information.

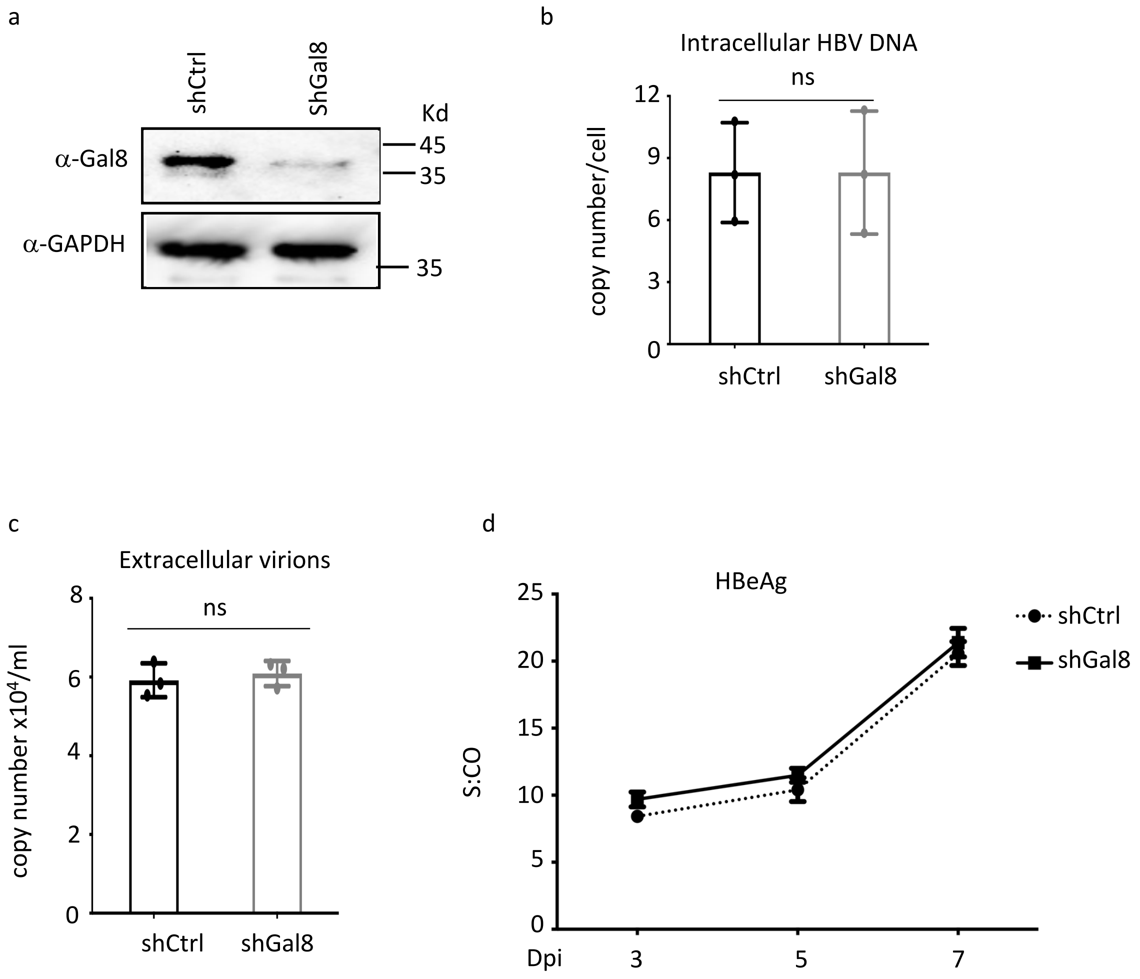

Supplementary Fig. 3. Galectin 8 is not involved in the regulation of HBV. a, Immunoblot analysis of indicated proteins from cellular extract of *galectin 8*-knockdown HepG2-NTCP (shGal8) and control cells (shCtrl). **b, c,** Cells were infected with HBV. HBV DNA from infected cells (b) or extracellular virions (c) were analyzed by qPCR (n = 3 biological replicates). ns: non significance. **d**, Medium levels of HBeAg from infected cells (n = 3 biological replicates). S:CO: signal-to-cutoff ratio. Dpi: day post infection. Data are means ± SD. Statistical significance in (b, c) is determined by a two-sided unpaired t-test. Source data for b,c,d are provided as Source Data file. Source data for a are provided as supplementary information.

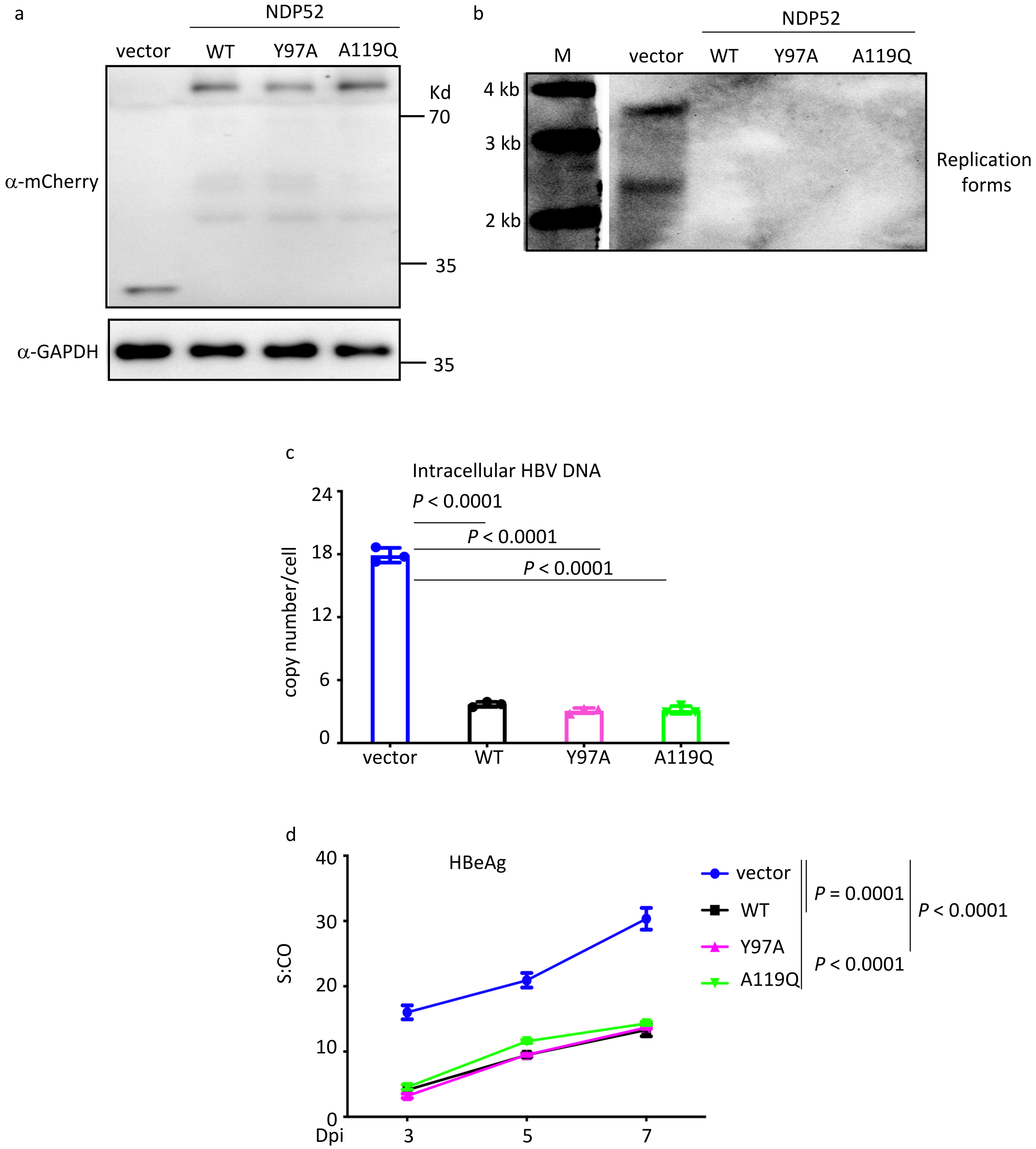

Supplementary Fig. 4. Mutations in NDP52 abolishing trimeric complex formation with FIP200 and NAP1/SINTBAD do not affect NDP52 activity on HBV. a, Immunoblot analysis of indicated proteins from cellular extract of $NDP52^{HepG2\text{-}NTCPKO}$ restored with mCherry tag vector (vector), mCherry-tagged wild-type NDP52 (WT), mCherry-tagged $NDP52_{Y97A}$ (Y97A), mCherry-tagged $NDP52_{A119Q}$ (A119Q) cells. **b**, Indicated cells were infected with HBV. Core DNA was purified and analyzed by Southern blot using an HBV DNA probe. M: DNA marker. **c**, Quantitative PCR of HBV DNA from infected cells (n = 3 biological replicates). **d**, Medium levels of HBeAg from infected cells (n = 3 biological replicates). S:CO: signal-to-cutoff ratio. Dpi: day post infection. Data are means ± SD. Statistical significance in (c, d) is determined by a two-sided unpaired t-test. Source data for c,d are provided as Source Data file. Source data for a,b are provided as supplementary information.

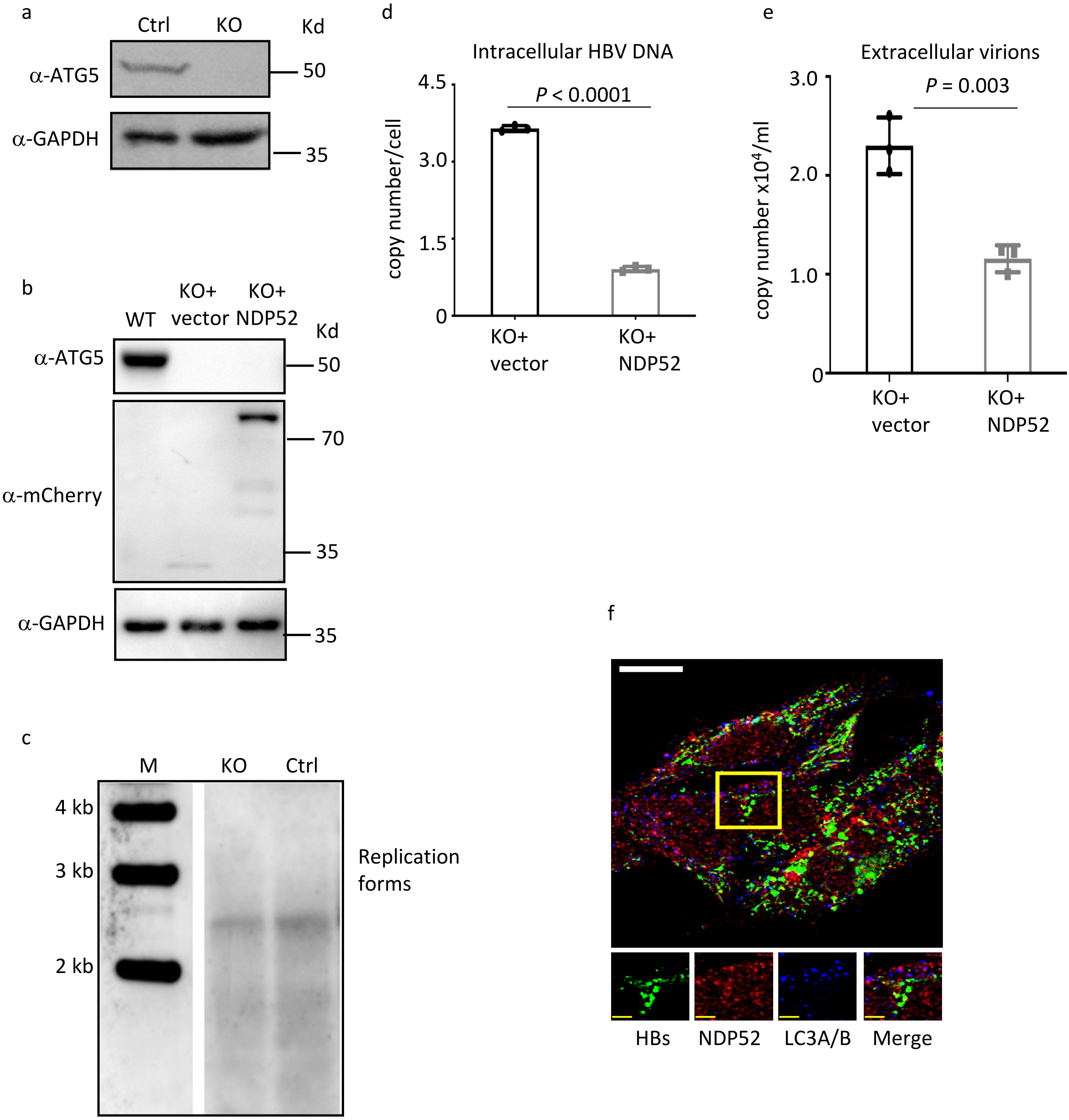

Supplementary Fig. 5. ATG5 is dispensable for NDP52-medaited viral degradation. a, Immunoblot analysis of indicated proteins from cellular extract of CRISPR control $ATG5^{HepG2\text{-}NTCPWT}$ (Ctrl) and $ATG5^{HepG2\text{-}NTCPKO}$ (KO) cells. **b**, Immunoblot analysis of indicated proteins from cellular extract of $ATG5^{HepG2\text{-}NTCPWT}$ (WT) and $ATG5^{HepG2\text{-}NTCPKO}$ transduced with either lentivirus vector (KO+vector) or expressing mCherry-tagged NDP52 (KO+NDP52). **c**, CRISPR control $ATG5^{HepG2\text{-}NTCPWT}$ (Ctrl) and $ATG5^{HepG2\text{-}NTCPKO}$ (KO) cells were infected with HBV. Core DNA was purified and detected by Southern blot analysis using an HBV DNA probe. M: DNA marker. **d**, **e**, Quantitative PCR of HBV DNA extracted from infected cells (d) or extracellular virions (e) ($n = 3$ biological replicates). **f**, HepAD38 cells were coimmunostained for viral envelope proteins (HBs), NDP52 and LC3. The scale bar is 10 μm for full cell images, 2.5 μm for zoomed images. Data are means $\pm$ SD. Statistical significance in (d, e) is determined by a two-sided unpaired t-test. Source data for d,e are provided as Source Data file. Source data for a,b,c are provided as supplementary information.

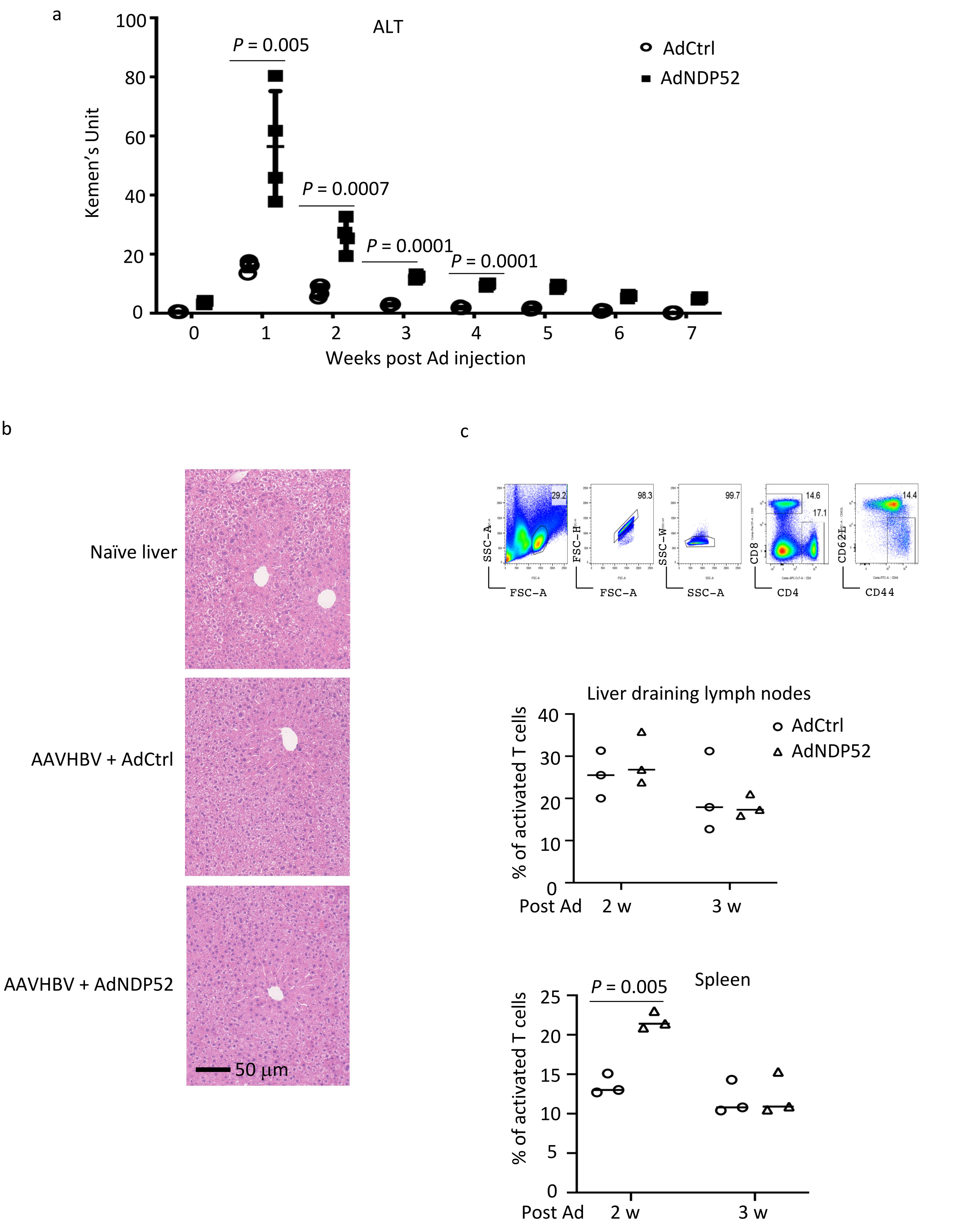

Supplementary Fig. 6. NDP52 suppresses HBV *in vivo*. a, 10-week-old male naïve mice were injected either with adenovirus vector (AdCtrl) or expressing human NDP52 (AdNDP52). Serum levels of alanine aminotransferase (ALT) were measured (n = 4 animals). **b**, H&E staining of indicated livers. **c**, Lymphocytes from liver draining lymph nodes and spleens were analyzed for $CD4^{+}CD44^{+}CD62L^{-}$ T cells by flow cytometry 2 and 3 weeks post recombinant adenovirus injection (n = 3 animals). Data are means ± SD. Statistical significance in (a, c) is determined by a two-sided unpaired t-test. Source data for a,c are provided as Source Data file.

Source file Sup Fig. 1b

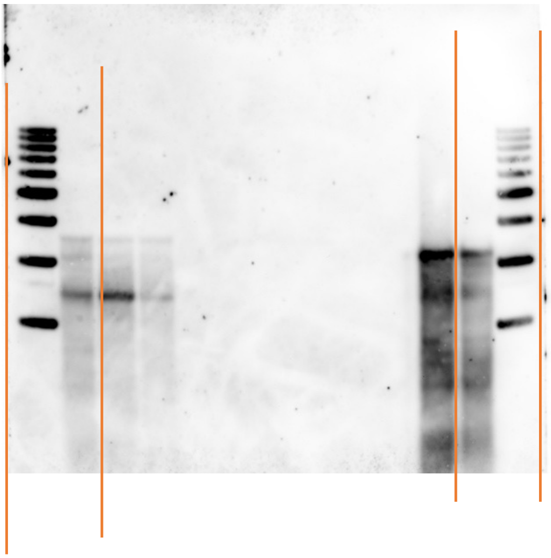

Source file Sup Fig. 2a

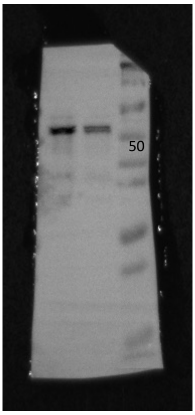

Anti-NDP52

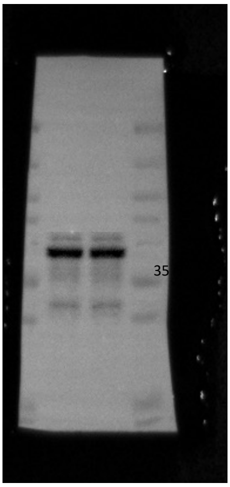

Anti-GAPDH

Source file Sup Fig. 2b

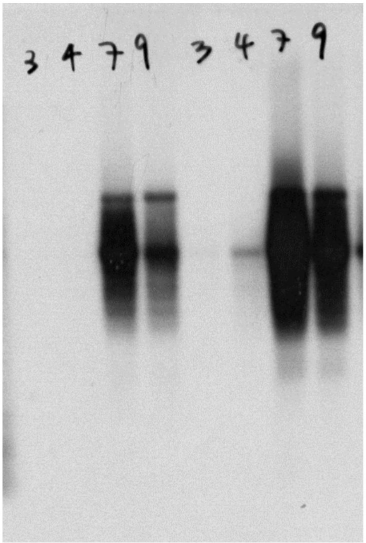

Source file Sup Fig. 2e

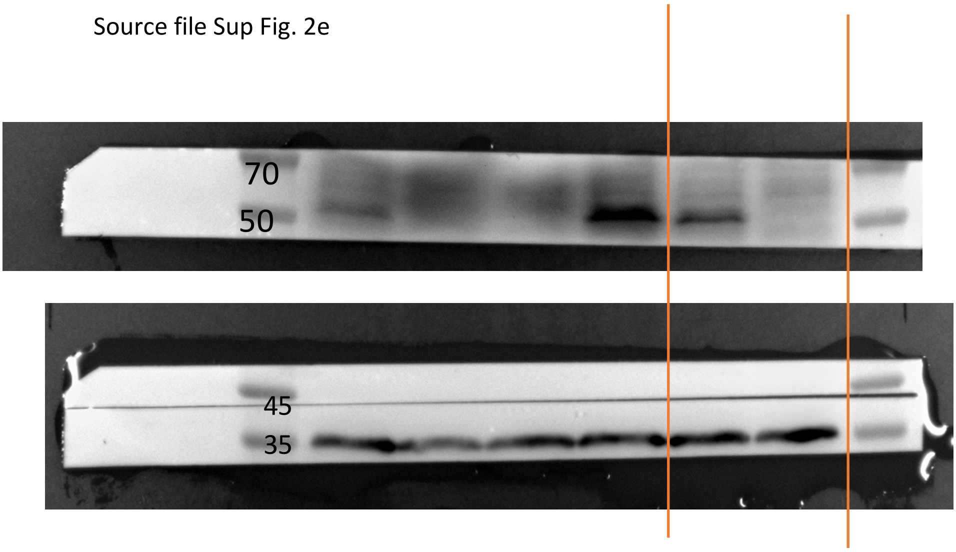

Anti-NDP52

Anti-GAPDH

Source file Sup Fig. 3a

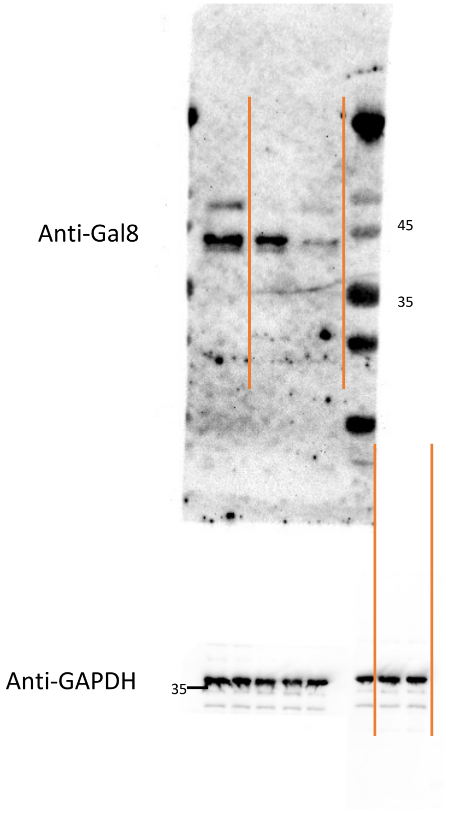

Source file Sup Fig. 4a

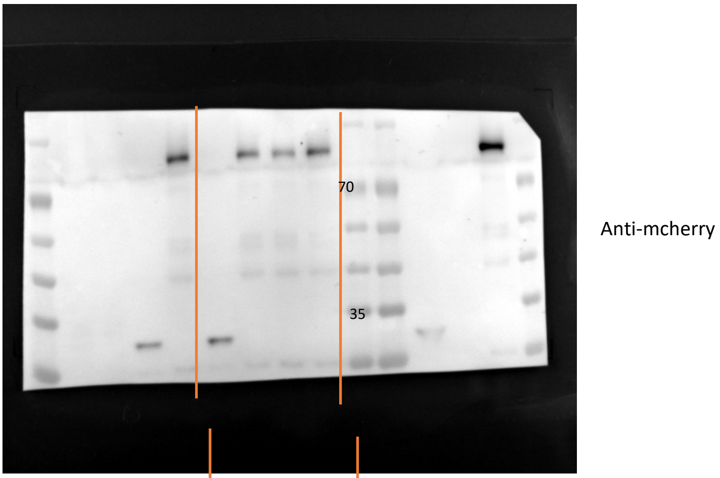

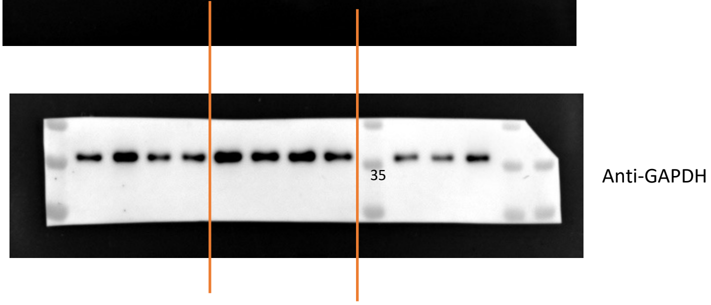

Source file Sup Fig. 4b

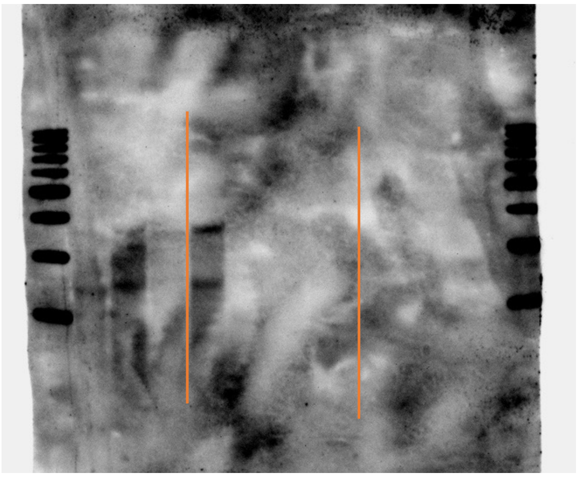

Source file Sup Fig. 5a

Anti-ATG5

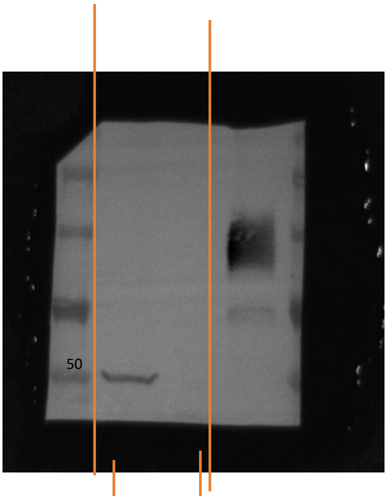

Anti-GAPDH

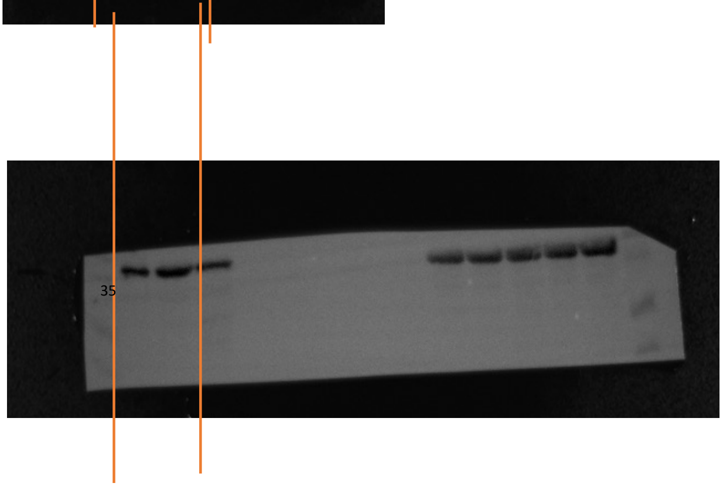

Source file Sup Fig. 5b

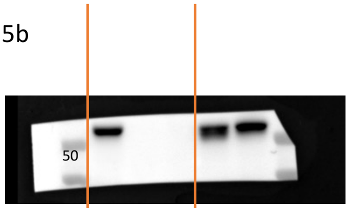

Anti-ATG5

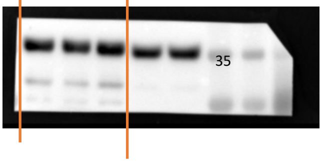

Anti-GAPDH

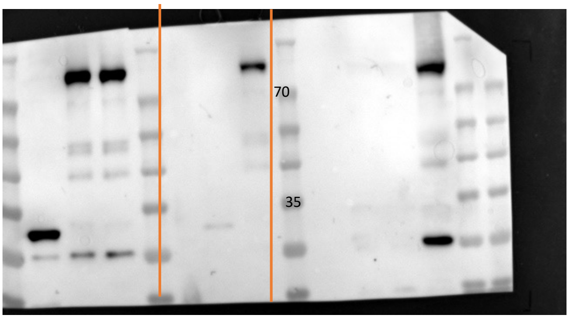

Anti-mcherry

Source file Sup Fig. 5c

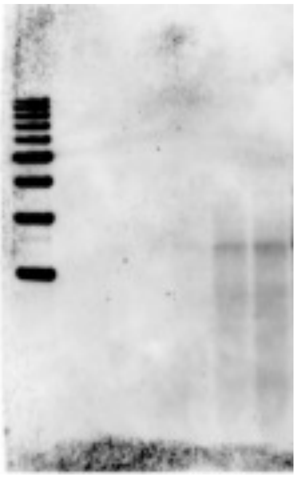

Supplement: Supplementary file 1 — Suuplementary information [file 41467_2023_44201_MOESM1_ESM.pdf]
