# Supplementary material for: NDP52 mediates an antiviral response to hepatitis B virus infection through Rab9-dependent lysosomal degradation pathway
Source: Nat Commun. 2023 Dec 19;14:8440. doi: 10.1038/s41467-023-44201-2 (PMC10730550; doi:10.1038/s41467-023-44201-2)
Supplement: Supplementary file 3 — Reporting Summary [file 41467_2023_44201_MOESM3_ESM.pdf]

nature portfolio

Corresponding author(s): Yu Wei

Last updated by author(s): Nov 30, 2023

Reporting Summary

Nature Portfolio wishes to improve the reproducibility of the work that we publish. This form provides structure for consistency and transparency in reporting. For further information on Nature Portfolio policies, see our Editorial Policies and the Editorial Policy Checklist.

Statistics

For all statistical analyses, confirm that the following items are present in the figure legend, table legend, main text, or Methods section.

n/a	Confirmed	
☐	☒	The exact sample size (*n*) for each experimental group/condition, given as a discrete number and unit of measurement
☐	☒	A statement on whether measurements were taken from distinct samples or whether the same sample was measured repeatedly
☐	☒	The statistical test(s) used AND whether they are one- or two-sided<br>*Only common tests should be described solely by name; describe more complex techniques in the Methods section.*
☒	☐	A description of all covariates tested
☒	☐	A description of any assumptions or corrections, such as tests of normality and adjustment for multiple comparisons
☐	☒	A full description of the statistical parameters including central tendency (e.g. means) or other basic estimates (e.g. regression coefficient) AND variation (e.g. standard deviation) or associated estimates of uncertainty (e.g. confidence intervals)
☐	☒	For null hypothesis testing, the test statistic (e.g. *F*, *t*, *r*) with confidence intervals, effect sizes, degrees of freedom and *P* value noted<br>*Give P values as exact values whenever suitable.*
☒	☐	For Bayesian analysis, information on the choice of priors and Markov chain Monte Carlo settings
☒	☐	For hierarchical and complex designs, identification of the appropriate level for tests and full reporting of outcomes
☐	☒	Estimates of effect sizes (e.g. Cohen's *d*, Pearson's *r*), indicating how they were calculated

Our web collection on statistics for biologists contains articles on many of the points above.

Software and code

Policy information about availability of computer code

Data collection	Data collection did not involve the use of software or code
Data analysis	All statistical analyses were performed using GraphPad Prism 9 software (GraphPad, San Diego, CA, USA). Pearson's correlation coefficient was used for quantifying colocalization. Flowcyto data were analyzed with Flowjo software (BD Bioscience).

For manuscripts utilizing custom algorithms or software that are central to the research but not yet described in published literature, software must be made available to editors and reviewers. We strongly encourage code deposition in a community repository (e.g. GitHub). See the Nature Portfolio guidelines for submitting code & software for further information.

Data

Policy information about availability of data

All manuscripts must include a data availability statement. This statement should provide the following information, where applicable:

- Accession codes, unique identifiers, or web links for publicly available datasets
- A description of any restrictions on data availability
- For clinical datasets or third party data, please ensure that the statement adheres to our policy

All of the data generated and analyzed during this study are included in this article.

Research involving human participants, their data, or biological material

Policy information about studies with human participants or human data. See also policy information about sex, gender (identity/presentation), and sexual orientation and race, ethnicity and racism.

Reporting on sex and gender	not applicable
Reporting on race, ethnicity, or other socially relevant groupings	not applicable
Population characteristics	not applicable
Recruitment	not applicable
Ethics oversight	not applicable

Note that full information on the approval of the study protocol must also be provided in the manuscript.

Field-specific reporting

Please select the one below that is the best fit for your research. If you are not sure, read the appropriate sections before making your selection.

[x] Life sciences [ ] Behavioural & social sciences [ ] Ecological, evolutionary & environmental sciences

For a reference copy of the document with all sections, see nature.com/documents/nr-reporting-summary-flat.pdf

Life sciences study design

All studies must disclose on these points even when the disclosure is negative.

Sample size	For in vivo experiment, we wanted to follow up HBsAg, HBeAg and ALT for 7 weeks after AdNDP52 treatment and to analyze immune cells 2 weeks and 3 weeks post AdNDP52. 9 animals/group were used at time 0, 3 animals were sacrificed at week 2 and week 3. Sample size was determined considering ethical use of animals in scientific research, limits in animal housing capacity, the number of animals that can be handled in one experiment, availability of reagents (AAVHBV, AdNDP52), and the need for it to offer sufficient statistical power.
Data exclusions	No data exclusions.
Replication	At least two independent studies were done.
Randomization	No randomization methods were used. Since all animals had the same age, similar weight, same sex (male) and showed similar levels of HBsAg and HBeAg at week 0, we believed there was no a real confounder that would impact the outcome of the experiments.
Blinding	The investigators knew group allocations in animal facility.

Reporting for specific materials, systems and methods

We require information from authors about some types of materials, experimental systems and methods used in many studies. Here, indicate whether each material, system or method listed is relevant to your study. If you are not sure if a list item applies to your research, read the appropriate section before selecting a response.

Materials & experimental systems

n/a	Involved in the study	
[ ]	[x]	Antibodies
[ ]	[x]	Eukaryotic cell lines
[x]	[ ]	Palaeontology and archaeology
[ ]	[x]	Animals and other organisms
[x]	[ ]	Clinical data
[x]	[ ]	Dual use research of concern
[x]	[ ]	Plants

Methods

n/a	Involved in the study	
[x]	[ ]	ChIP-seq
[ ]	[x]	Flow cytometry
[x]	[ ]	MRI-based neuroimaging

Antibodies

Antibodies used	GST (Sigma Aldrich, G7781), Flag (Sigma, F7425), HA (Sigma Aldrich, H6908), GAPDH (Abmart, M20006L), GFP (Sigma Aldrich, G1546), NDP52 (Abcam, ab68588), mcherry (ABclonal, AE002), LC3A/B (Cell Signaling Technology, 12741S), HBV preS2 (Abcam, ab8635), ATG5 (Cell Signaling Technology, 12994S), Rab9A (Cell Signaling Technology, 5118), Lamp2 (Santa Cruz, sc-18822), Galectin 8 (Santa

	Cruz, sc-377133). HBsAg (Abcam, ab32914), calcoco2 (Santa Cruz, sc-376540), CD8 PerCP/Cy5.5 (Biolegend, BLE100734), CD44 Monoclonal Antibody (IM7) FITC (Invitrogen, 11-0441-82), CD62L MEL-14 APC (eBioscience, 17-0621-82).
Validation	All the antibodies are commercial antibodies which are validated by suppliers.

Eukaryotic cell lines

Policy information about cell lines and Sex and Gender in Research

Cell line source(s)	HepG2: ATCC #HB-8065, HEK293T: ATCC#CRL-3216, HepAD38 cells were kindly provided by Dr. Christoph Seeger (Fox Chase Cancer Center, Philadelphia, USA). Huh7 cells, established by Sato, J., and Nakabayshi, H., in 1982, have been widely distributed in research laboratories woldwide since then.
Authentication	None of the cell lines used were authenticated.
Mycoplasma contamination	All the cell lines were tested negative for mycoplasma contamination.
Commonly misidentified lines (See ICLAC register)	No commonly misidentified lines were used.

Animals and other research organisms

Policy information about studies involving animals; ARRIVE guidelines recommended for reporting animal research, and Sex and Gender in Research

Laboratory animals	C57BL/6J male mice were used.
Wild animals	not applicable
Reporting on sex	The findings in this study apply to both sex. Sex was considered in study design. Only male animals were used in this study, because men infected with hepatitis B virus are 6 times more likely than women to develop chronic hepatitis B.
Field-collected samples	not applicable
Ethics oversight	All animal experiments were performed in accordance with institutional guidelines and approved by the Institutional Animal Care and Use Committee of the Institut Pasteur of Shanghai, Chinese Academy of Sciences.

Note that full information on the approval of the study protocol must also be provided in the manuscript.

Flow Cytometry

Plots

Confirm that:

- [x] The axis labels state the marker and fluorochrome used (e.g. CD4-FITC).
- [x] The axis scales are clearly visible. Include numbers along axes only for bottom left plot of group (a 'group' is an analysis of identical markers).
- [x] All plots are contour plots with outliers or pseudocolor plots.
- [x] A numerical value for number of cells or percentage (with statistics) is provided.

Methodology

Sample preparation	Mice were euthanazied at week 2 and week 3 post AdNDP52, splenocytes and liver draining lymph nodes were collected and used for flow cytometry analysis. Single-cell suspensions were prepared and incubated with 2.4G2 antibody before staining with fluorochrome-conjugated antibodies.
Instrument	Fortessa Flow Cytometer (BD Biosciences)
Software	Flowjo software (BD Bioscience)
Cell population abundance	Cell population abundance is shown as % of parent population.
Gating strategy	Lymphocyte population > CD4 positive, CD44 positive and CD62L negative.

- [x] Tick this box to confirm that a figure exemplifying the gating strategy is provided in the Supplementary Information.
